# Phytochemical Analysis and Antioxidant Evaluation of the Ethanolic Extract of the Leaves of Abutilon indicum

**DOI:** 10.7759/cureus.47703

**Published:** 2023-10-26

**Authors:** Mannala Sunil, T Vedavijaya, Karuna Sree P, Suresh Babu Sayana

**Affiliations:** 1 Department of Pharmacology, Meenakshi Academy of Higher Education and Research, Chennai, IND; 2 Department of Pharmacology, Meenakshi Ammal Dental College and Hospital, Chennai, IND; 3 Department of Pharmacology, All India Institute of Medical Sciences Guwahati, Guwahati, IND; 4 Department of Pharmacology, Government Medical College and General Hospital, Suryapet, IND

**Keywords:** pharmacology, antioxidant efficacy, therapeutic attributes, luteolin, abutilon indicum

## Abstract

Background: In Indian traditional medicine, the Abutilon indicum plant, colloquially known as "Country mallow" or "Thuthi", has been vouched for its efficacy in treating conditions such as bronchitis and diabetes. The study aimed to explore the chemical constituents and antioxidant strength of the ethanolic extracts derived from the leaves of this plant (ELEAI).

Objectives: To qualitatively pinpoint the phytochemicals in the ethanolic extract of abutilon indicum leaves (ELEAI), utilize high-performance thin layer chromatography (HPTLC) to quantitatively analyze the identified compounds within the ELEAI, and gauge its antioxidant capability through the DPPH method, benchmarking the outcomes against the recognized standard, ascorbic acid.

Methods: Abutilon indicum leaves, originating from Telangana, were authenticated by taxonomists at Osmania University. After cleaning and drying, the leaves were powdered. A mixture of ethanol and water (70:30 ratio) was then used to extract the compounds in a Soxhlet extractor for a duration of 72 hours at a temperature of 60°C. The liquid extract was subsequently evaporated to form a light-brown powder, which was stored at 20°C under shade for later use.

Results: Preliminary analyses indicated that ELEAI was rich in both primary and secondary metabolites. Luteolin, a known phytochemical, was quantitatively confirmed in the extract using HPTLC. Impressively, the DPPH assay highlighted ELEAI's remarkable antioxidant capabilities.

Conclusion: Abutilon indicum showcases notable therapeutic potential with its rich phytochemical content and strong antioxidant activity, making it a promising candidate for further pharmacological research and natural drug formulation.

## Introduction

Throughout history, plants have been a wellspring of ethnomedicine, with approximately 14%-18% of higher plants having documented medicinal uses [1]. Remarkably, about 74% of pharmacologically active plants have been unveiled through investigations into their ethnomedicinal applications [2]. Within plants, a distinction exists between primary and secondary metabolites. Primary metabolites encompass essential compounds like sugars, amino acids, proteins, nucleic acids, and polysaccharides [3]. In contrast, secondary plant metabolites constitute a diverse array of chemical compounds synthesized via specialized metabolic pathways branching off from primary ones. These secondary metabolites comprise a diverse range of chemical compounds, including glycosides, flavonoids, phenolics, alkaloids, saponins, terpenes, lipids, and carbohydrates. These secondary metabolites have been associated with a wide spectrum of advantageous effects, establishing the scientific foundation for their utilization in traditional medicine across various historical societies [4,5]. These compounds exhibit properties such as antibiotic, antifungal, and antiviral activities, serving to protect plants from pathogens. For instance, phenolic compounds, a subset of secondary metabolites, showcase a diverse range of bioactivities [5].

Secondary metabolites obtained from medicinal plants exhibit noteworthy biological properties, encompassing hypoglycemic, anti-diabetic, antioxidant, anti-microbial, anti-inflammatory, anti-carcinogenic, antimalarial, anticholinergic, and anti-leprosy activities. As a result, the active compounds found in medicinal plants have garnered significant attention for their multifaceted applications [6,7]. These secondary metabolites not only serve as precursors and prototypes for drug development but also act as valuable pharmacological probes. They play a pivotal role in global drug discovery efforts and can even serve as foundational blueprints for the development of synthetic drugs or as essential building blocks for semi-synthetic drug production [8].

One particularly intriguing medicinal plant is Abutilon indicum, commonly known as Tutturubenda, which belongs to the Malvaceae family. This plant thrives in the hot regions of India and has an extensive history in traditional medicine, particularly in Siddha medicine, where it is employed to treat conditions such as jaundice, piles, ulcers, and leprosy [9,10]. Abutilon indicum has also been linked to analgesic effects and potential impacts on fertility. In certain regions, the juice extracted from its leaves is combined with Allium cepa extract to address jaundice, supported by hepatoprotective studies conducted on experimental animals [11].

Given this backdrop, this study embarks on a qualitative and quantitative phytochemical investigation with the goal of identifying potential bioactive compounds in the ethanolic leaf extract of abutilon indicum (ELEAI) and evaluating its antioxidant activity. The study aimed to qualitatively and quantitatively analyze the phytochemical composition of the ethanolic leaf extract of abutilon indicum (ELEAI). Through preliminary phytochemical tests and high-performance thin layer chromatography (HPTLC), the research sought to identify diverse bioactive compounds and determine their concentrations. Additionally, the study evaluated ELEAI's antioxidant potential by comparing it to ascorbic acid using the DPPH method. These objectives laid the foundation for a comprehensive exploration of ELEAI's phytochemical profile and its potential health benefits.

## Materials and methods

Plant material and authentication: The study was conducted at the Department of Pharmacology, Malla Reddy Institute of Medical Sciences, Hyderabad, India. Plant samples were meticulously gathered from various locations in Telangana, and their authenticity was confirmed by a taxonomist at Osmania University's Botanical Department, Telangana, India. Chemicals and reagents used in the study were procured from the Sigma-Aldrich Company.

Preliminary phytochemical tests: A battery of preliminary phytochemical tests was conducted on the ethanolic leaves extract of abutilon indicum (ELEAI), following the methods described by Kokate CK [5] and Ansari SH [6]. The summarized tests are presented in Table 1.

**Table 1:** The qualitative phytochemical analysis of the ethanolic leaves extract of Abutilon indicum (ELEAI) "+" Symbol: This symbol typically denotes a positive test result, indicating the presence of the particular phytochemical in the ethanolic leaves extract of abutilon indicum (ELEAI) for the corresponding test. For example, under the alkaloids row, Dragendroff’s Test, Wagner's Test, and Mayer's Test all show a "+", suggesting that alkaloids were detected in the extract using these tests. "−" Symbol: This symbol typically denotes a negative test result, indicating the absence of the particular phytochemical in the ELEAI for the corresponding test. For example, under the Tannins row, both the Gallotannins test and the Ellagitannins test show a "−", suggesting that these types of tannins were not detected in the extract using these tests.

Test	Test Name	ELEAI
Alkaloids	Dragendroff’s Test Wagner's Test Mayer's Test	+ + +
Amino acids	Millon’s Test Ninhydrin Test	+
Carbohydrates	Molisch Test Barfoed Test Seliwanoff Test	+
Flavonoids	Shinoda Test Alkaline reagent Test Zinc Hydrochloride test	+ +
Phenolics compounds	Ferric Chloride Test	+
Tannins	Gallotannins Test Ellagitannins Test	− −
Steroids & triterpenoids	Salkowaski Test Sulfur Powder Test	− +
Saponins	Foam Test	+

High-performance thin layer chromatography (HPTLC) procedure

Luteolin standard solution preparation: To create a luteolin standard solution, 10 mg of luteolin was dissolved in 10 ml of methanol, resulting in a concentration of 1000 µg/ml. From this solution, 1 ml was further diluted with methanol to yield a 100 µg/ml solution. Subsequently, 10 µl of this diluted solution was carefully applied to a thin layer chromatography (TLC) plate.

Sample preparation: The sample under investigation was dissolved in methanol.

Procedure: The following steps were followed for the chromatographic analysis:

Standard and sample solutions (10 µl each) were applied to the TLC plate as bands with a width of 6 mm and a 5 mm gap between bands. This application was done using a 100-µl sample syringe from Hamilton (Bonaduz, Switzerland). A pre-coated silica gel aluminum plate 60 F254 (5 cm × 10 cm) with a thickness of 250 µm (from E. MERCK, Darmstadt, Germany) was used as the stationary phase. The slit dimensions were set at 5 mm × 0.45 mm, and the scanning speed was maintained at 20 mm/sec. Chromatography was conducted in a twin-trough glass chamber (10 cm × 10 cm) using a mobile phase consisting of n-Hexane and ethyl acetate in a 5:5 v/v ratio. The mobile phase was allowed to saturate the chamber for 15 minutes to ensure optimal conditions. The chromatogram run length was 8 cm, and the development time took approximately 15 minutes. Following the chromatographic separation, the TLC plates were dried using a hair dryer. Densitometric scanning was performed at a wavelength of 400 nm using a CAMAG thin-layer chromatography scanner. The scanner was operated with WINCATS software version 1.4.2. This comprehensive procedure enabled the separation and quantification of luteolin in both the standard and sample solutions using HPTLC.

Antioxidant activity assessment (DPPH method)

Principle: The DPPH radical is capable of accepting hydrogen from an antioxidant. The degree of antioxidant activity is directly correlated with the reduction in DPPH concentration within the test sample. Requirements are ascorbic acid, DPPH (1,1-diphenyl-2-picrylhydrazyl), extract of *A. indicum*.

Preparation of solutions

Various working solutions of ascorbic acid were prepared, ranging from concentrations of 5 µg/mL to 3000 µg/mL. Similarly, working solutions of the extract and its different fractions were prepared, covering a concentration range of 5 µg/mL to 3000 µg/mL. A DPPH solution was created by dissolving 3 mg of DPPH in 100 mL of methanol. A methanolic solution (1 ml of 0.2 mM) of DPPH was mixed with 1 mL each of the extract and L-ascorbic acid solutions at varying concentrations. As a control, a DPPH solution with the addition of 1 mL of methanol was utilized. All prepared solutions were kept in darkness for a duration of 30 minutes. The absorbance of these solutions was measured at 517 nm using a UV spectrophotometer, ensuring the readings were adjusted to less than one. The color transitioned from purple to yellow as the DPPH was reduced by the hydrogen provided by the antioxidants.

The capacity of plant extracts to scavenge DPPH free radicals was determined using the following formula: as % Inhibition = [(Ac - As) / Ac] x 100, where: Ac represents the absorbance of the control, As represents the absorbance of the sample. This formula quantified the extent of inhibition or scavenging of DPPH radicals by the plant extract, providing a measure of its antioxidant activity.

Statistical analysis

Results are expressed as mean ± SD. The data were subjected to a one-way analysis of variance (ANOVA), followed by a Dunnett's t test. Ethical approval for all experimental procedures was obtained from the Institutional Animal Ethics Committee of Malla Reddy Institute of Medical Sciences, Hyderabad, India (MRIMS-IAEC-CCSEA, 02/2023).

## Results

In our qualitative phytochemical analysis detailed in Table 1, we successfully identified a diverse range of phytochemical compounds within the ethanolic leaf extract of abutilon indicum (ELEAI). These compounds included alkaloids, amino acids, carbohydrates, flavonoids, phenolic compounds, tannins, triterpenoids, and saponins, demonstrating the richness and complexity of ELEAI's phytochemical composition.

Moving to the quantitative estimations outlined in Table 2, ELEAI exhibited notable quantities of specific phytochemicals.

**Table 2:** Quantitative estimation of the phytochemicals present in the ethanolic leaf extract of Abutilon indicum ELEAI: Ethanolic leaf extract of abutilon indicum

Test (mg/g dry plant extract)	ELEAI
Total flavonoid content	8.32±1.24
Total alkaloids content	6.41±2.06
Total phenolics content	11.46±1.96

The total phenolic content was measured at 11.46 mg GAE/g extract, indicating a substantial presence of phenolic compounds, which are known for their antioxidant properties. Additionally, the total flavonoid content was determined to be 8.32 mg QE/g extract, underscoring the flavonoid-rich nature of ELEAI. Furthermore, the extract contained a significant total alkali content of 6.41 mg CE/g, suggesting the potential bioactivity associated with alkaloid compounds.

Our quantitative phytochemical analysis also included the identification of luteolin in ELEAI using the HPTLC method. Chromatograms clearly indicated the presence of luteolin in ELEAI, with an Rf value of 0.38. This finding is noteworthy, as luteolin is a bioactive compound associated with various health benefits. Finally, our investigation into the antioxidant activity of ELEAI, compared to the standard compound ascorbic acid, is presented in Table 3.

**Table 3:** Antioxidant activity of ethanolic leaf extract of abutilon indicum (ELEAI) and standard compound (ascorbic acid).

Concentration	Superoxide Radical Scavenging Activity (%)		NO Radical Scavenging Activity (%)		H2O2 Radical Scavenging Activity (%)	
(µg/mL)	EEAI	Ascorbic Acid	EEAI	Ascorbic Acid	EEAI	Ascorbic Acid
5 µg/mL	20.16 ± 2.62	13.25 ± 0.32	19.66 ± 1.42	21.35 ± 2.12	20.56 ± 1.62	28.35 ± 1.12
10 µg/mL	22.04 ± 0.12	19.12 ± 1.3	24.55 ± 0.49	29.5 ± 0.82	24.51 ± 0.98	39.54 ± 2.82
25 µg/mL	39.53 ± 1.23	31.21 ± 2.22	35.23 ± 1.61	40.65 ± 2.18	27.75 ± 1.28	44.66 ± 0.98
50 µg/mL	48.46 ± 1.43	38.26 ± 2.62	46.76 ± 3.54	48.75 ± 2.33	32.87 ± 2.21	47.41 ± 3.10
75 µg/mL	53.12 ± 6.31	50.26 ± 3.12	54.63 ± 1.32	57.13 ± 2.65	39.51 ± 0.78	49.34 ± 0.97
100 µg/mL	62.19 ± 1.28	58.14 ± 4.23	61.32 ± 2.9	66.49 ± 2.14	52.85 ± 1.87	57.57 ± 0.18
150 µg/mL	70.56 ± 0.12	63.26 ± 2.62	65.71 ± 2.23	71.83 ± 1.87	61.25 ± 2.32	67.35 ± 1.07
200 µg/mL	72.24 ± 1.68	71.25 ± 1.92	70.85 ± 1.67	81.43 ± 0.82	66.87 ± 2.42	68.67 ± 2.62
250 µg/mL	86.24 ± 1.26	80.26 ± 1.72	72.57 ± 1.65	86.51 ± 1.32	73.76 ± 3.23	78.91 ± 1.82
300 µg/mL	95.39 ± 2.72	90.25 ± 3.12	79.52 ±1.72	90.64 ± 0.23	80.43 ± 2.08	86.18 ±1.14
IC50 (µg/mL)	76.16 ± 1.28	79.89 ± 1.1 ,8	70.12 ± 1.59	67.70 ± 1.54	97.43 ± 2.56	89.38 ± 0.45

The results illustrated a dose-dependent quenching effect on DPPH radicals by ELEAI, indicating strong DPPH scavenging capacity. Notably, ELEAI exhibited an IC50 value of 89.49 ± 1.23 mg/mL, suggesting its potential as an effective antioxidant agent. Remarkably, this IC50 value closely resembled that of the standard ascorbic acid (IC50 99.36 ± 1.89 µg/mL), underscoring the antioxidant potential of ELEAI.

In summary, our findings highlight the diverse phytochemical composition of ELEAI, including the presence of specific bioactive compounds such as luteolin. Moreover, ELEAI demonstrated strong antioxidant properties, making it a promising candidate for further exploration of its potential health benefits.

## Discussion

Medicinal plants have been a source of diverse secondary metabolites, many of which exhibit valuable biological activities with therapeutic potential. In this comprehensive study, we performed a qualitative analysis of various phytochemical compounds present in the ethanolic leaves extract of abutilon indicum (ELEAI). These compounds encompassed a wide spectrum, including phenols, reducing sugars, flavones, saponins, alkaloids, proteins, glycosides, and triterpenoids, all of which have been associated with a myriad of bioactivities [12,13].

The presence of such bioactive compounds in medicinal plants like abutilon indicum often makes them attractive for therapeutic applications. These compounds have been reported to contribute to hepatoprotection and may offer protection against various chronic diseases, including cancer, diabetes, and cardiovascular disorders, as documented in a study by Choi et al. in 2015 [14].

Our comprehensive investigation confirmed the presence of a diverse array of phytochemicals within ELEAI extracts, underscoring its potential as a valuable natural resource for medicinal purposes. The identified phytochemicals included flavonoids, alkaloids, phenolic compounds, tannins, saponins, steroids, glycosides, and terpenoids. Notably, these compounds exhibited substantial antioxidant activity, suggesting their capacity to counteract oxidative stress and protect against cellular damage caused by free radicals [15,16]. Furthermore, ELEAI displayed high quantities of total phenolics (11.46 mg GAE/g extract), total flavonoids (8.32 mg QE/g extract), and total alkaloids (6.41 mg CE/g extract). These findings indicate the richness of ELEAI in these bioactive compounds, further reinforcing its potential therapeutic value [17].

The remarkable presence of such phytochemicals in ELEAI opens up possibilities for various beneficial effects, particularly when administered to counteract hepatotoxic agents induced by drugs and chemicals. The hepatoprotective potential of ELEAI could be attributed to its antioxidant properties and the presence of specific phytochemicals known for their hepatoprotective effects [18,19].

The study limitations include focus on a specific geographic region (Telangana, India) for the collection of Abutilon indicum leaves, which may limit the generalizability of the results to other regions with varying environmental conditions. Moreover, while the DPPH assay provided valuable insights into the antioxidant capacity of ELEAI, further in-depth studies are required to understand its potential therapeutic effects in vivo and its safety profile. 

## Conclusions

Our qualitative phytochemical analysis of ELEAI unveiled the presence of both primary metabolites (amino acids and carbohydrates) and secondary metabolites (alkaloids, flavonoids, phenolic compounds, tannins, terpenoids, saponins, steroids, glycosides, and fatty acids). Our quantitative analysis further demonstrated significant levels of total phenolics, flavonoids, and alkaloids in ELEAI. Moreover, using HPTLC, we successfully identified and quantified luteolin in ELEAI, with an Rf value of 0.38, potentially serving as a chemical marker for standardizing Abutilon indicum.

ELEAI exhibited strong DPPH scavenging capacity, closely resembling the standard ascorbic acid. These insights are crucial for setting standards for this medicinal plant, with its phytochemicals showing promise in various therapeutic applications, including anti-inflammatory, antioxidant, and hepatoprotective properties.

## References

[1] Kumar M, Kumar Patel M, Kumar N, Bajpai AB, Siddique KH (2021). Metabolomics and molecular approaches reveal drought stress tolerance in plants. Int J Mol Sci.

[2] Wawrosch C, Zotchev SB (2021). Production of bioactive plant secondary metabolites through in vitro technologies-status and outlook. Appl Microbiol Biotechnol.

[3] Yoganarasimhan SN, Chelladurai V (2000). Medicinal Plants of India. https://indianmedicine.eldoc.ub.rug.nl/2589/.

[4] Johri RK, Pahwa GS, Sharma SC, Zutshi U (1991). Determination of estrogenic/antiestrogenic potential of antifertility substances using rat uterine peroxidase assay. Contraception.

[5] Ansari SH (2006). Essentials of Pharmacognosy. New Delhi: Birla publications.

[6] Kokate CK (1994). Practical Pharmacognosy.

[7] Wu X, Dhanasekaran S (2020). Protective effect of leaf extract of Abutilon indicum on DNA damage and peripheral blood lymphocytes in combating the oxidative stress. Saudi Pharm J.

[8] Abcha I, Criado P, Salmieri S (2019). Edible Rhus tripartita fruit as source of health-promoting compounds: characterization of bioactive components and antioxidant properties. Eur Food Res Technol.

[9] Bendary E, Francis RR, Ali HMG, Sarwat MI, El Hady S (2013). Antioxidant and structure-activity relationships (SARs) of some phenolic and anilines compounds. Ann Agric Sci.

[10] Das K, Khan MS, Namratha N (2019). Comparative phytochemical screening, elemental content and chromatographic evaluation for detection and quantification of polyphenolic compounds for strong antioxidant activity of various extracts of Abutilon indicum (Link) Sweet leaves. Ann Phytomed.

[11] Gomaa AAR, Samy MN, Desoukey SY (2018). Phytochemistry and pharmacological activities of genus Abutilon: a review (1972-2015). J Adv Biomed PharmSci.

[12] Petrovska BB (2012). Historical review of medicinal plants' usage. Pharmacogn Rev.

[13] Erb M, Kliebenstein DJ (2020). Plant secondary metabolites as defenses, regulators, and primary metabolites: the blurred functional trichotomy. Plant Physiol.

[14] Choi MK, Kim HG, Han JM, Lee JS, Lee JS, Chung SH, Son CG (2015). Hepatoprotective Effect of Terminalia chebula against t-BHP-Induced Acute Liver Injury in C57/BL6 Mice. Evid Based Complement Alternat Med.

[15] Rahman MM, Rahaman MS, Islam MR (2021). Role of phenolic compounds in human disease: current knowledge and future prospects. Molecules.

[16] Rady I, Bloch MB, Chamcheu RN (2018). Anticancer properties of graviola (Annona muricata): A comprehensive mechanistic review. Oxid Med Cell Longev.

[17] Vadnere GP, Pathan AR, Kulkarni BU, Singhai AK (2013). Abutilon indicum linn: a phytopharmacological review. Int J Res Pharm Chem.

[18] Ahmed M, Amin S, Islam M, Takahashi M, Okuyama E, Hossain CF (2000). Analgesic principle from Abutilon indicum. Pharmazie.

[19] Porchezhian E, Ansari SH (2000). Effect of liquid extract from fresh Abutilon indicum leaves and Allium cepa bulbs on paracetamol and carbontetrachloride induced hepatotoxicity. Pharmazie.

